# MiR93-5p inhibits chondrocyte apoptosis in osteoarthritis by targeting lncRNA CASC2

**DOI:** 10.1186/s12891-019-3025-y

**Published:** 2020-01-13

**Authors:** Yun Sun, Simiao Kang, Shuyan Pei, Changmin Sang, Yijun Huang

**Affiliations:** 1 Department of Orthopaedics, Jiujiang University Clinical Medical College·Jiujiang University Hospital, No.57 Xunyang East Road, Jiujiang City, jiangxi Province 332000 People’s Republic of China; 2 Department of Orthopaedics, Harbin Children’s Hospital, Harbin City, Heilong Jiang Province 150000 People’s Republic of China

**Keywords:** Osteoarthritis, CASC2, miR-93-5p, Chondrocyte, Apoptosis

## Abstract

**Background:**

It has been reported that miR-93-5p and long non-coding RNA (lncRNA) Cancer Susceptibility 2 (CASC2) play opposite roles in regulating chondrocyte apoptosis, indicating the possible interaction between them. This study aimed to investigate the interaction between miR-93-5p and lncRNA CASC2 in chondrocyte apoptosis, which plays critical roles in osteoarthritis (OA).

**Methods:**

The interaction between CASC2 and miR-93-5p was analyzed by dual luciferase assay and overexpression experiments. Levels of CASC2 and miR-93-5p in plasma sample from OA patients and healthy controls were measured by RT-qPCR. The roles of CASC2 and miR-93-5p in regulating the apoptosis of chondrocyte induced by LPS were analyzed by cell apoptosis assay.

**Results:**

Through bioinformatics analysis we observed the potential interaction between CASC2 and miR-93-5p, which was confirmed by dual luciferase assay. In OA patients, miR-93-5p was downregulated, while CASC2 was upregulated, and they were inversely correlated. LPS treatment led to downregulated miR-93-5p and upregulated CASC2. Overexpression of miR-93-5p led to the downregulated CASC2 in chondrocytes. Under LPS treatment, CASC2 overexpression promoted the apoptosis of chondrocyte. MiR-93-5p overexpression played an opposite role and attenuated the effects of CASC2 overexpression.

**Conclusion:**

MiR-93-5p was downregulated in OA may inhibit LPS-induced chondrocyte apoptosis by targeting lncRNA CASC2.

## Background

Osteoarthritis (OA), also known as “wear and tear” arthritis or degenerative joint disease, mainly affect elderly with hips, knees and hands as the major affects sites [1]. OA is a major cause of chronic pain and disability, and a heavy burden on public health [2]. In some developed countries, the direct medical cost and indirect cost by reduced productivity causes the loss of 1 to 2.5% of the gross domestic product [3]. Aging, being female gender, obesity and occupational joint loading are the major risk factors for OA [4]. However, molecular pathogenesis of OA remains elusive [5, 6], leading to the difficulties in the development of novel therapeutic approaches.

Previous studies have shown that genetic factors are critical players in the molecular pathogenesis of OA [7, 8]. Some signaling pathways, such as WNT signaling, have been proven to be potential targets for the development of targeted therapies [9]. Besides protein-coding genes, non-coding RNAs (ncRNAs), such as miRNAs and long (>200 nt) ncRNAs (lncRNAs), also regulate gene expression at multiple levels to participate in OA [10]. For instance, miR-93-5p inhibits the apoptosis of chondrocyte by targeting TCF4, thereby improving OA [11]. In contrast, lncRNA CASC2 promotes the apoptosis of chondrocytes to aggregate the conditions of OA [12]. The opposite functions of miR-93-5p and CASC2 indicate the potential interactions between them in OA. In effect, our bioinformatics analysis also revealed the potential interaction between miR-93-5p and CASC2. This study was therefore carried out to analyze the potential interaction between miR-93-5p and CASC2 in OA.

## Methods

### OA patients and controls

Research subjects of this study included 60 OA patients (20 males and 40 females, 55 to 71 years, 63.0 ± 5.3 years) and 60 healthy controls (20 males and 40 females, 56 to 71 years, 63.3 ± 5.1 years) who were admitted to Jiujiang University Clinical Medical College, Jiujiang University Hospital between March 2016 and June 2019. This study passed the review of Ethics Committee of aforementioned hospital. OA patients’ inclusion criteria: 1) patients who were diagnosed for the first time; 2) no therapies were initiated before admission. OA patients’ exclusion criteria: 1) other clinical disorders were diagnosed; 2) recurrent OA. The 60 OA patients included 27 cases of stage III and 33 cases of stage IV. According to the affected sites, there were 31 knee-affected cases and 29 hip-affected cases. The diagnosis of OA was performed through conventional methods, such as joint fluid analysis and X-ray imaging. Controls were enrolled to match the age and gender distributions of OA patients. All participants were informed of experimental design of this project. All patients and controls signed informed consent.

### Synovial fluid

Before the initiation of therapies, extraction of synovial fluid from the affected sites was performed on all patients. To match OA patients, extraction of synovial fluid from knee was performed on 31 controls and extraction from hip was performed on the rest 29 cases. A liquid nitrogen sink was used to store the samples before use.

### Chondrocytes and transient transfections

Primary chondrocytes (402OA-05A) from an OA adult were purchased from Sigma-Aldrich (USA) and were cultivated under conditions of 37 °C with 5% CO_2_ in Chondrocyte Growth Medium (PromoCell). Cells were harvested at passage 5 to 7 to perform following experiments.

CASC2 expression vectors were constructed using pcDNA3.1 vector (Sangon) as backbone. Synthesis of miR-93-5p mimic and miRNA negative control (NC) was performed by Sangon. Lipofectamine 2000 Transfection Reagent (Invitrogen) was used to transfect 50 Nm miRNA (miRNA NC as NC group) or 10 Nm vector (empty vector as NC group) into 106 cells. Control© cells were untransfected cells. The interval between following experiments and transfections was 24 h.

### Luciferase reporter assay

CASC2 full length cDNA was cloned into pGL3 plasmids (Promega). Through the aforementioned methods, cells were transfected with CASC2 vector+ miR-93-5p mimic or CASC2 vector+miRNA NC. Luciferase activity was measured by Dual Luciferase Reporter Assay Kit (Promega Corporation) using cells harvested at 24 h post-transfection.

### RNA samples and qPCR

RNAiso Plus kit (Takara) was used to extract total RNA from synovial fluid specimens and in vitro cultivated cells. To harvest miRNAs, 85% was used to precipitate and wash RNA samples. To remove genomic DNA, all RNA samples were digested with DNA eraser at 37 °C for 1 h. In cases of LPS treatment, chondrocytes were treated with LPS at a concentration of 0, 200, 500 and 100 ng for 24 h before use.

Total RNA reverse transcriptions (RTs) were performed using PrimeScript RT Master Mix (Takara) with total RNA as template to synthesize cDNA samples. With cDNA samples as template, qPCR mixtures were prepared using SYBR Premix Ex TaqTM II (Takara, Japan) to measure the expression levels of CASC2. 18S rRNA was used as the endogenous control of CASC2.

To measure the expression levels of mature miR-93-5p, polyadenylation, RTs and qPCR mixtures were performed using All-in-One™ miRNA qRT-PCR Reagent Kit (Genecopoeia). U6 was used as the endogenous control of miR-93-5p.

PCR reactions were performed in 3 replicates and fold-changes of gene expression were calculated using 2^−ΔΔCt^ method.

### Cell apoptosis analysis

Cells were harvested at 24 h post-transfection and cell suspensions (3 × 10^5^/ml) were prepared using non-serum cell culture medium. Cells were seeded onto a six-well plate with 2 ml cell suspension per well, followed by the addition of 1 μg/mL LPS. Following cell culture under aforementioned methods for 24 h, Annexin V-FITC Apoptosis Detection Kit (Thermo Fisher Scientific) was used to detect apoptotic cells. FACSCalibur flow cytometer was used to perform flow cytometer and data were analyzed by CellQuest software (BD Biosciences).

### Western-blot

RIPA solution (Invitrogen) was used to was used to isolate proteins from chondrocytes. Proteins were denatured in boiling water for 10 min and were separated by 10% SDS-PAGE gel electrophoresis. PVDF membranes were used to transfer proteins and blocking was performed in fat-free milk (5% in PBS) at room temperature for 90 min. Primary antibodies included rabbit anti-human cleaved caspase 3 (ab49822, 1:1000; Abcam) and GAPDH (ab9485, 1: 1000, Abcam) at 4 °C for 15 h, followed by incubation with IgG-HRP goat anti-rabbit secondary antibody (MBS435036, 1:1000, MyBioSource) at room temperature for 2 h. Signals were produced using Amersham ECL Western Blotting Detection Reagent (GE Healthcare) and were normalized using Image J v1.46 software.

### Statistical analysis

Means ± standard deviation (SD) was used to express the data of 3 independent biological replicates involved in each experiment. Unpaired t test was used to compare 2 groups. Exploration of differences among multiple groups was performed using ANOVA (one-way) and Tukey test. Correlations were analyzed by linear regression. *p* < 0.05 indicated statistically significant differences.

## Results

### CASC2 can directly interact with miR-93-5p

**Fig. 1:**
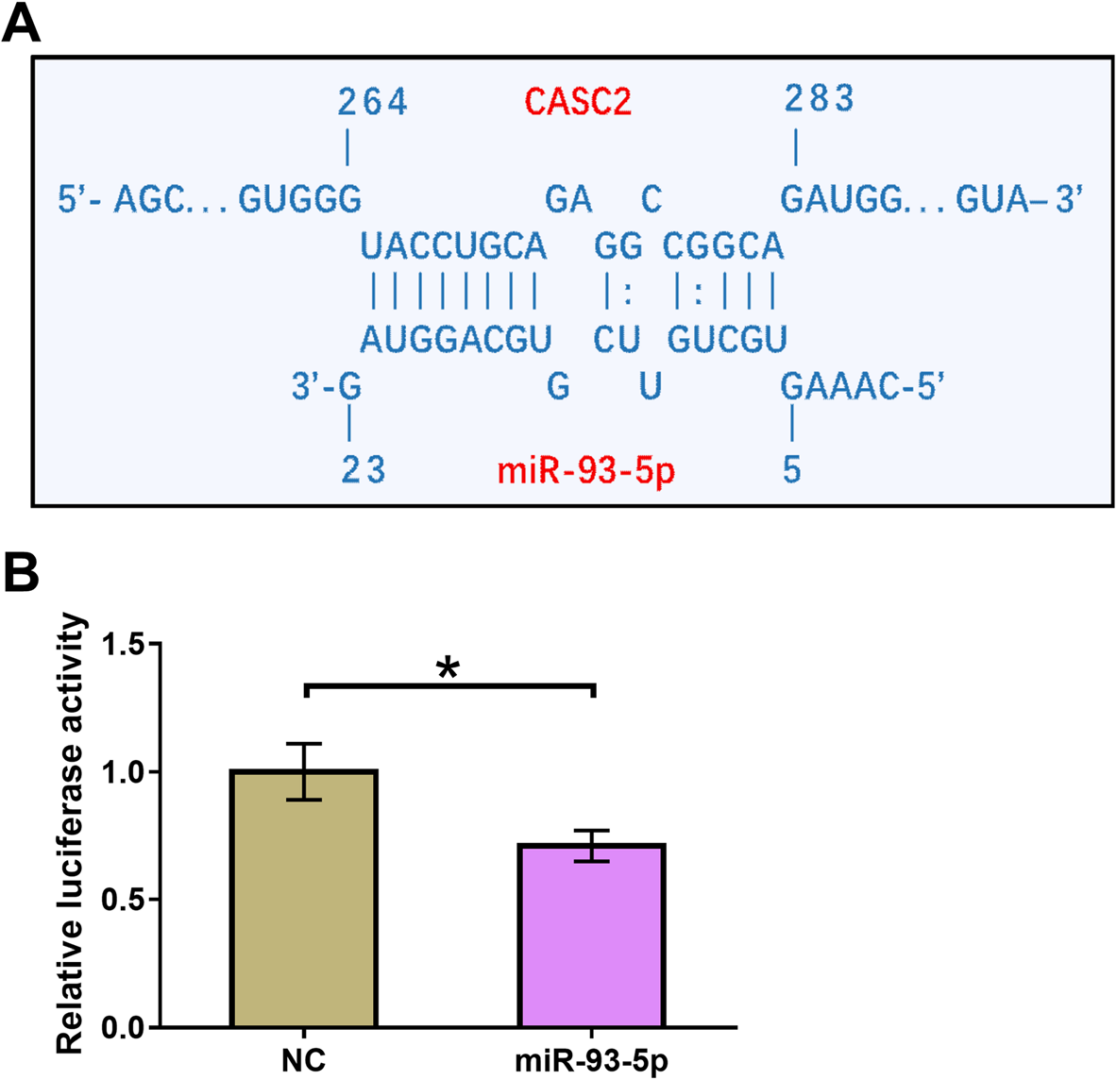
CASC2 can directly interact with miR-93-5p. The interaction between CASC2 and miR-93-5p was predicted using an online RNA interaction program named IntaRNA (http://rna.informatik.uni-freiburg.de/IntaRNA/Input.jsp). It was observed that CASC2 and miR-93-5p can form strong base pairing (A). Dual luciferase reporter assay was performed by transfecting CASC2 vector and miRNA NC or CASC2 vector and miR-93-5p mimic into chondrocytes. Relative luciferase activity was compared by unpaired t test. Experiments were repeated 3 times and mean values were presented.*, *p* < 0.05

The interaction between CASC2 and miR-93-5p was predicted using an online RNA interaction program named IntaRNA (http://rna.informatik.uni-freiburg.de/IntaRNA/Input.jsp). It was observed that CASC2 and miR-93-5p can form strong base pairing (Fig. 1a). Dual luciferase reporter assay was performed to further analyze the interaction between CASC2 and miR-93-5p. Comparing to chondrocytes transfected with CASC2 vector and miRNA NC, cells transfected with CASC2 vector and miR-93-5p mimic showed significantly lower relative luciferase activity (Fig. 1b, *p* < 0.05).

### MiR-93-5p and CASC2 were inversely correlated in synovial fluid

**Fig. 2:**
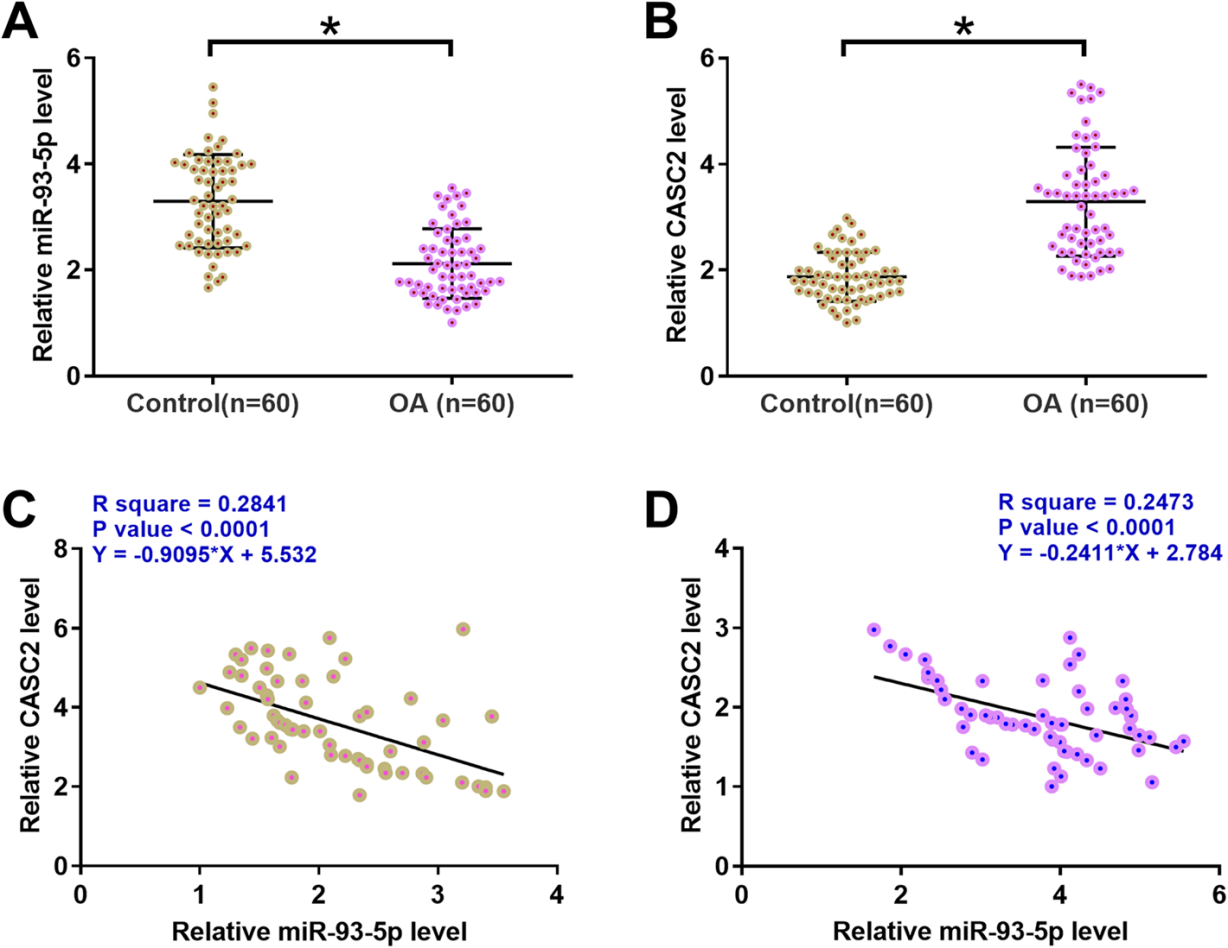
MiR-93-5p and CASC2 were inversely correlated in synovial fluid**.** Levels of miR-93-5p (A) and CASC2 (B) in synovial fluid from both OA (*n* = 60) and healthy controls (*n* = 60) were measured using qPCR and compared by unpaired t test. PCR reactions were performed 3 times and mean values were presented. *, *p* < 0.05. Linear regression was performed to analyze the correlation between expression levels of CASC2 and miR-93-5p across both OA (C) and control (D) synovial fluid samples

Levels of miR-93-5p and CASC2 in synovial fluid from both OA (*n* = 60) and healthy controls (n = 60) were measured using qPCR and compared by unpaired t test. It was observed that, comparing to controls, miR-93-5p was significantly downregulated (Fig. 2a, *p* < 0.05), while CASC2 was significantly upregulated (Fig. 2b, *p* < 0.05) in OA group. Linear regression analysis showed that expression levels of CASC2 were significantly and inversely correlated with expression levels of miR-93-5p across both OA (Fig. 2c) and control (Fig. 2d) synovial fluid samples.

### Overexpression of miR-93-5p led to the downregulated CASC2 in chondrocytes

**Fig. 3:**
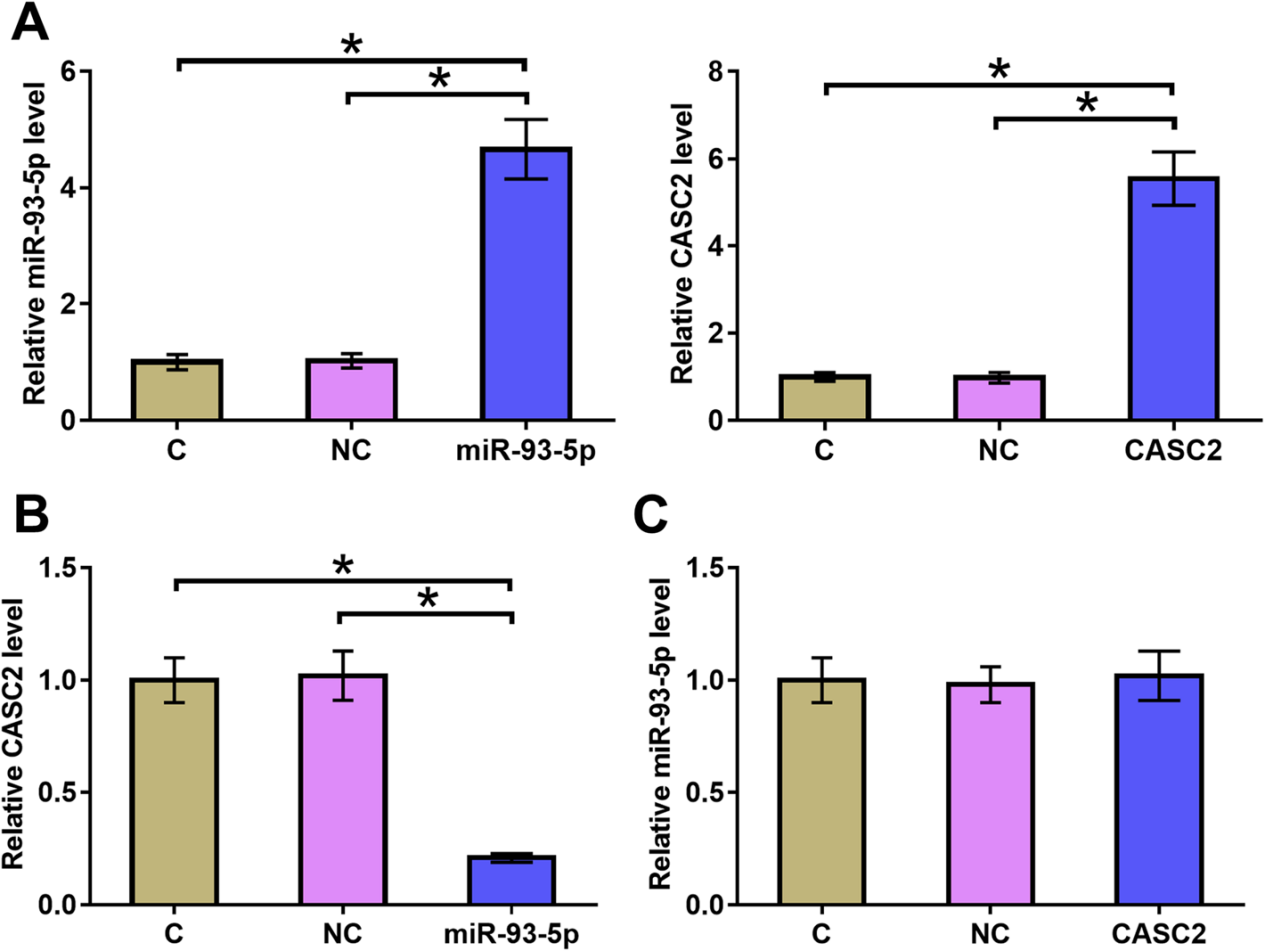
Overexpression of miR-93-5p led to the downregulated CASC2 in chondrocytes. Chondrocytes were transfected with miR-93-5p mimic and CASC2 expression vector. Overexpression of miR-93-5p and CASC2 was confirmed by qPCR at 24 h post-transfection (**a**). The effects of miR-93-5p overexpression on CASC2 (**b**) and the effects of CASC2 overexpression on miR-93-5p (**c**) were also analyzed by qPCR. Experiments were repeated 3 times and mean values were presented.*, *p* < 0.05

Chondrocytes were transfected with miR-93-5p mimic and CASC2 expression vector. Overexpression of miR-93-5p and CASC2 was confirmed by qPCR at 24 h post-transfection (Fig. 3a, *p* < 0.05). Comparing to C and NC groups, overexpression of miR-93-5p led to downregulated CASC2 (Fig. 3b, *p* < 0.05). In contrast, CASC2 overexpression failed to significantly affect the expression of miR-93-5p (Fig. 3c, *p* < 0.05).

### MiR-93-5p overexpression inhibited the apoptosis of chondrocytes induced by LPS through CASC2

**Fig. 4:**
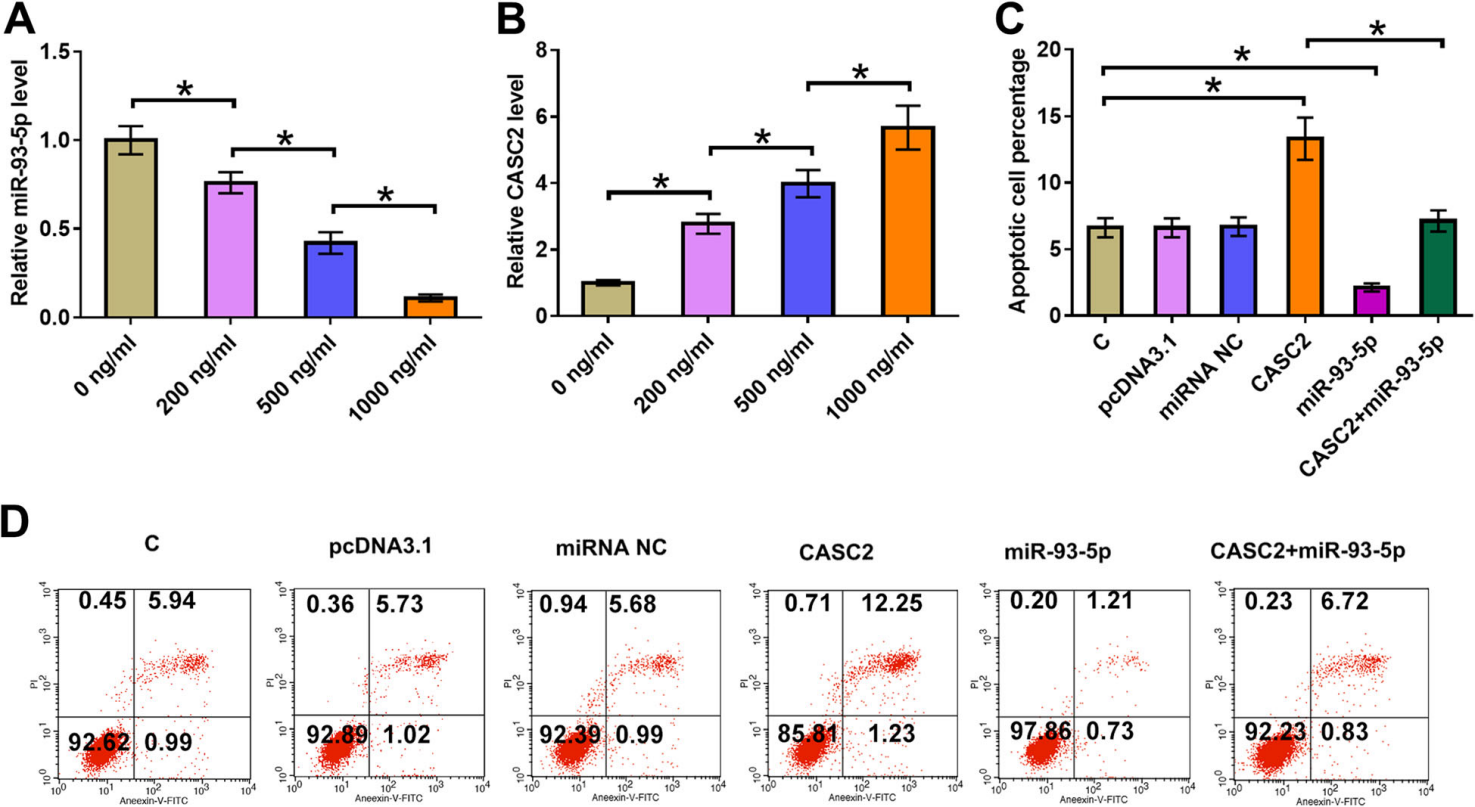
MiR-93-5p overexpression inhibited the apoptosis of chondrocytes induced by LPS through CASC2. Chondrocytes were treated with LPS at a concentration of 0, 200, 500 and 100 ng for 24 h, followed by the measurement of miR-93-5p (**a**) and CASC2 (**b**) by qPCR. Cell apoptosis assay was performed to analyze the effects of CASC2 and miR-93-5p CASC2 on apoptosis of chondrocytes induced by LPS (**c**). Experiments were repeated 3 times and mean values were presented.*, *p* < 0.05

**Fig. 5:**
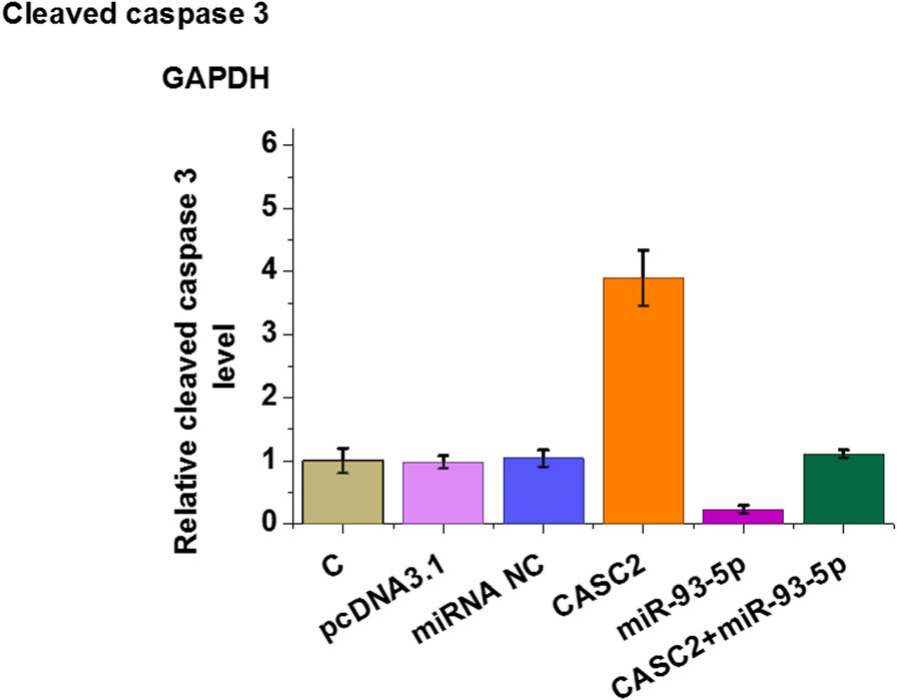
MiR-93-5p overexpression suppressed the production of cleaved caspase 3 in chondrocytes through CASC2. Western blot was performed to analyze the effects of CASC2 and miR-93-5p CASC2 on the production of cleaved caspase 3 in chondrocytes treated by LPS (C). Experiments were repeated 3 times and representative images were presented.*, *p* < 0.05

Chondrocytes were treated with LPS at a concentration of 0, 200, 500 and 100 ng for 24 h, followed by the measurement of miR-93-5p and CASC2 by qPCR. It was observed that LPS treatment led to downregulated miR-93-5p (Fig. 4a, *p* < 0.05) and upregulated CASC2 (Fig. 4b, *p* < 0.05) in a dose-dependent manner. Cell apoptosis assay was performed to analyze the effects of CASC2 and miR-93-5p CASC2 on apoptosis of chondrocytes induced by LPS. Comparing to C group, CASC2 overexpression promoted the apoptosis of chondrocyte. MiR-93-5p overexpression played an opposite role and attenuated the effects of CASC2 overexpression (Fig. 4c, *p* < 0.05). Consistently, CASC2 overexpression led to increased level of cleaved caspase 3. MiR-93-5p overexpression played an opposite role and attenuated the effects of CASC2 overexpression on the production of cleaved caspase 3 (Fig. 5, *p* < 0.05).

## Discussion

This study mainly explored the interaction between miR-93-5p and CASC2 and their roles in OA. We first characterized CASC2 as a downstream target of miR-93-5p.

CASC2 is a well-characterized tumor suppressive lncRNAs in many different types of cancers [13, 14]. CASC2 participate in cancer biology mainly by regulating cancer cell behaviors, such as inhibiting cell proliferation and promoting cell apoptosis [13, 14]. The development of OA leads to cartilage damage, while the only cells in healthy cartilage are chondrocytes [15]. In effect, the apoptosis of chondrocytes plays a major role in the pathogenesis of OA [15]. A recent study reported that CASC2 was upregulated in OA and promoted chondrocyte apoptosis to aggregate disease conditions [12]. Consistently, our study also observed increased apoptotic rate of chondrocytes under LPS treatment after CASC2 overexpression. Our data and previous studies showed that CASC2 may play similar roles in regulating the apoptosis of different types of cells to participate in different types of diseases.

Inhibition of chondrocyte apoptosis is considered as a promising target for the treatment of OA [16]. In a recent study, Xu et al. reported that miR-93-5p can target TCF4 to suppress cartilage degradation and the apoptosis of chondrocyte to improve the conditions of OA [11]. Our study used LPS-treated chondrocytes as the cell model of OA because LPS participates in diverse pathological processes involved in OA, ranging from inflammatory responses and cell apoptosis [17]. Consistent with previous studies, our study also showed the inhibitory effects of miR-93-5p on the apoptosis of chondrocytes induced by LPS.

The key finding of the present study is that miR-93-5p can directly target CASC2 to participate in the regulation of chondrocyte apoptosis. Therefore, miR-93-5p may have multiple downstream targets (TCF4 as aforementioned) in chondrocytes. Future studies are needed to explore other potential targets.

## Conclusion

In conclusion, miR-93-5p is downregulated in OA and can directly target CASC2 to inhibit LPS-induced apoptosis of chondrocytes.

## Data Availability

The analyzed data sets generated during the study are available from the corresponding author on reasonable request.

## Abbreviations

C: Control
CASC2: Cancer Susceptibility 2
GAPDH: Glyceraldehyde 3-phosphate dehydrogenase
lncRNA: long non-coding RNA
LPS: Lipopolysaccharides
NC: Negative control
OA: Osteoarthritis
RT: Reverse transcription
